# Inflammatory cell response to ultra-thin amorphous and crystalline hydroxyapatite surfaces

**DOI:** 10.1007/s10856-016-5814-2

**Published:** 2016-11-28

**Authors:** Louise Rydén, Omar Omar, Anna Johansson, Ryo Jimbo, Anders Palmquist, Peter Thomsen

**Affiliations:** 1 Department of Biomaterials, Institute of Clinical Sciences, Sahlgrenska Academy, University of Gothenburg, Box 412, Gothenburg, SE-405 30 Sweden; 2 BIOMATCELL, VINN Excellence Center of Biomaterials and Cell Therapy, Gothenburg, Sweden; 3 Department of Oral and Maxillofacial Surgery and Oral Medicine, Faculty of Odontology, Malmö University, Malmö, Sweden

## Abstract

It has been suggested that surface modification with a thin hydroxyapatite (HA) coating enhances the osseointegration of titanium implants. However, there is insufficient information about the biological processes involved in the HA-induced response. This study aimed to investigate the inflammatory cell response to titanium implants with either amorphous or crystalline thin HA. Human mononuclear cells were cultured on titanium discs with a machined surface or with a thin, 0.1 μm, amorphous or crystalline HA coating. Cells were cultured for 24 and 96 h, with and without lipopolysaccharide (LPS) stimulation. The surfaces were characterized with respect to chemistry, phase composition, wettability and topography. Biological analyses included the percentage of implant-adherent cells and the secretion of pro-inflammatory cytokine (TNF-α) and growth factors (BMP-2 and TGF-β1). Crystalline HA revealed a smooth surface, whereas the amorphous HA displayed a porous structure, at nano-scale, and a hydrophobic surface. Higher TNF-α secretion and a higher ratio of adherent cells were demonstrated for the amorphous HA compared with the crystalline HA. TGF-β1 secretion was detected in all groups, but without any difference. No BMP-2 secretion was detected in any of the groups. The addition of LPS resulted in a significant increase in TNF-α in all groups, whereas TGF-β1 was not affected. Taken together, the results show that thin HA coatings with similar micro-roughness but a different phase composition, nano-scale roughness and wettability are associated with different monocyte responses. In the absence of strong inflammatory stimuli, crystalline hydroxyapatite elicits a lower inflammatory response compared with amorphous hydroxyapatite.

## Introduction

Hydroxyapatite (HA) is a principal example of biomaterials with osteoconductive and osteoinductive capacity [1]. Hydroxyapatite closely resembles the mineral phase of bone and has demonstrated superior bone ingrowth, osseointegration and bone bonding [2–4]. However, due to its limited mechanical properties, HA has been mainly applied as a coating for load-bearing applications, such as dental implants and un-cemented arthroplasties [5–9]. Various coating technologies have been evaluated, resulting in different surface characteristics [10–12]. One important aspect of the HA coating is its thickness. Thin HA coatings have been found to remain stable during bone matrix mineralization [13], while thicker HA coatings might crack and/or delaminate, with a risk of particulate release [14]. Thin HA coatings have also been shown to promote the same osteogenic response as thicker coatings [8]. Furthermore, experimental studies have shown promising results using thin sputtered HA coatings (0.1 μm thickness) on titanium implants [5, 8]. However, there is insufficient knowledge about the biological factors that could contribute to the improved bone response to the thin HA coating. One important aspect is the way the thin HA coating influences fundamental biological processes, such as the inevitable inflammatory cell response. In addition, it is important to determine whether an intentional change in the HA phase composition, e.g., amorphous vs. crystalline, would be sensed/perceived differently by the inflammatory cells.

Titanium implants with thin HA coatings elicit a less intense inflammatory reaction when compared with a titanium surface in a soft-tissue model [15]. However, although some trends were observed, the study did not reveal any major difference in cytokine secretions when comparing the thin crystalline and amorphous HA coatings. As a cell-specific effect cannot be excluded, it is possible that variations in the thin HA crystallinity produce different effects on the monocytes/macrophages, which are major cells in the early inflammatory response around biomaterials. Monocytes/macrophages are among the first cells to be found at the implant surface and are probably also the most abundant ones [16, 17]. Due to their early appearance and their versatile nature, they have been thought to play a pivotal role in both successful integration and failure [18]. Macrophages secrete multiple cytokines and mediators, which have a major influence on the tissue regeneration process [19]. These mediators include pro-inflammatory cytokines, such as tumor necrosis factor-alpha (TNF-α), and anti-inflammatory mediators, such as interleukin-10 (IL-10). Moreover, monocytes/macrophages are able to secrete some growth factors, including transforming growth factor-beta (TGF-β) [20], which performs multifunctional roles affecting different cell types and biological processes.

It has also been postulated that monocytes/macrophages are capable of producing a potent pro-osteogenic factor, the bone morphogenetic protein (BMP-2) [21], which suggests osteogenic potential for monocytes/macrophages. Interestingly, it has been shown that conditioned medium (CM) from monocytes/macrophages influences the osteogenic differentiation of mesenchymal stem cells [22]. However, in the latter study, the secretion of BMP-2 was not detected in the monocyte CM. It therefore remains to be explored whether or not monocytes/macrophages secrete osteoinductive factors, such as BMP-2, particularly in response to materials with osteoinductive potential, such as HA.

The aim of this study was to investigate the early monocyte/macrophage response to titanium implants with either amorphous or crystalline thin HA coatings and to compare this to a native machined titanium surface. One main scientific question is whether the response of the primary human monocytes/macrophage to the HA surfaces involves the triggered secretion of a multi-function growth factor, the TGF-β1, and/or a potent pro-osteogenic factor, the BMP-2.

## Materials and methods

### Implants and surface characterization

Titanium discs with a diameter of 15 mm and a thickness of 1 mm were machined from a rod of grade 2 titanium (Stupino Titanium Company, Russia). Two thirds of the discs were coated with 0.1 μm of hydroxyapatite (HA) (Ca_10_(PO_4_)_6_(OH)_2_) using radiofrequency (RF) magnetron sputtering, resulting in a thin amorphous HA coating. Half the amorphous HA-coated discs were subjected to heat treatment (600 °C), resulting in a thin crystalline HA coating. The discs were cleaned using 70 % ethanol in an ultrasonic bath for 30 s. All the discs were subsequently sterilized by *γ*-radiation, 1 × 25 Gy (IBA, Esbergærde, Denmark). The implants were further tested for endotoxin contamination and the analysis revealed no detectable levels (<0.006 EU/ml) at any of the implants using a Limulus quantitative chromogenic assay.

The different surfaces were characterized with regard to surface chemistry, phase composition, wettability and topography. The surface chemistry was analyzed by X-ray photoelectron spectroscopy (XPS) using a Quantum 2000 (Physical Electronics, Germany), equipped with a monochromatic Al Kα X-ray source, and time of flight-secondary ion mass spectroscopy (TOF-SIMS; Ion-TOF GmbH, Germany), equipped with a bismuth cluster ion gun and C60 gun. For XPS, the HA surfaces were sputter-cleaned for 5 s using Ar prior spectrum acquisition. Overview and high-resolution scans were then performed with regard to Ca2p, P2p and O1s. The Ca/P ratio was determined by measuring the areas under the Ca2p and P2p. The phase composition was assessed by X-ray diffraction using D5000 diffractometer (Siemens, Germany) with Cu Kα radiation for 2Θ 25–45. Water contact angle measurements were made using Fibro-DAT1100 (Fibro System AB, Stockholm, Sweden). A four-µl droplet of deionized water was put on the discs and the angle was measured at intervals for a total of 60 s (total of 130 measurements) in order to obtain a steady-state value.

The topographical aspects were analyzed with an optical interferometer (MicroXAM, Phase Shift, AZ, USA) using MapVue software (Meta MAP, Lexington, KY, USA). A Gaussian filter (filter size: 50 × 50 μm) was used to filter out waviness and vibration errors. For each implant type, 10 points were randomly selected and measured. The following parameters were evaluated; height descriptive parameter, *S*_a_ (arithmetic mean deviation of a surface); hybrid parameter, S_dr_ (the ratio between the developed surface area and a flat reference area); and functional parameter, S_ci_ (core retention fluid index). 3D images were reconstructed with MountainsMap 6.2 imaging software (Digital Surf, Paris, France). Scanning electron microscopy (SEM) was used for the evaluation of the morphology and submicron structure, using an Ultra 55 FEG SEM (Leo Electron microscopy, Zeiss, Oberkochen, Germany).

### Cell isolation

Peripheral blood mononuclear cells were separated from buffy coats, from six donors, using a method described by Pertoft [23]. Polyvinylpyrrolidone-coated silica gel (Percoll^TM^, Amersham Pharmacia Biotech AB, Sweden) was used as a gradient. Buffy coats were layered on top of 1.076 g/ml Percoll and centrifuged at 800 g for 30 min at room temperature. The layer with the mononuclear cells was then aspirated and washed twice in HBSS (without Ca^2+^ and Mg^2+^), which included centrifugation at 350 g for 5 min at 4 °C. The suspension of mononuclear cells was then layered on top of 1.064 g/ml Percoll and centrifuged at 400 g for 60 min at 4 °C. The mononuclear cells were aspirated and washed three times in HBSS. The cell viability after the separation was 97.5 ± 2.5 %, as determined by Trypan blue dye.

### Cell culture

The monocytes were re-suspended in RPMI 1640 supplied with 5 % FCS (fetal calf serum) and 1 % PEST (penicillin and streptomycin) at a concentration of 10^6^ cells/ml with a volume of 1 ml per well_._ The cells were seeded on the different surfaces in 24-well plates (NUNC^TM^, Nalge Nunc International, Denmark), in duplicate, with or without lipopolysaccharide (LPS; Escherichia coli, serotype 0127:B8, Sigma-Aldrich, USA) at concentration of 10 ng/ml. The plates were incubated for 24 h and 96 h at 37 °C with 5 % CO_2_ and 95 % humidity.

### Cytotoxicity

The cytotoxicity was evaluated after 24 and 96 h by measuring the activity of lactate dehydrogenase (LD) in the culture medium. The enzyme LD catalyzes the conversion of pyruvate to lactate by oxidizing NADH to NAD^+^. LD activity is measured spectrophotometrically at 340 nm and is correlated to the change in absorbance, which is due to the oxidation of NADH (C-Laboratory, Sahlgrenska University Hospital, Sweden).

### Cell counting

The number of cells was determined with NucleoCounter^TM^ (Chemometec, Allerød, Denmark). Both cells in the supernatant and cells adhering to the disc surface were counted, after which the ratio of the adherent cells to the supernatant cells was determined.

### ELISA measurements

The supernatants were collected after 24 and 96 h and centrifuged at 400 g for 5 min. The presence and concentration of different cytokines were determined using the enzyme-linked immunosorbent assay technique (ELISA; Quantikine^®^, R&D Systems, USA). The optical density was measured spectrophotometrically using an automatic plate reader (Spectra MAX plus, Molecular Devices, Crawley, UK). The optical density was read at 450 nm with the subtraction of readings at 570 nm in order to correct for plate imperfections. The supernatants were analyzed for the presence of tumor necrosis factor-alpha (TNF-α), transforming growth factor-beta 1 (TGF-β1) and bone morphogenetic protein-2 (BMP-2).

### Statistics

Statistical analyses were performed using non-parametric Friedman and Wilcoxon signed-rank tests. The analyses were performed in IBM SPSS^®^ Statistic software (SPSS, Inc., Chicago, USA), with *p* < 0.05 regarded as a statistically significant difference. The data are presented as the mean ± standard error of the mean.

## Results

### Implant surface characterization

The XPS analysis showed that the coated discs were composed of Ca, P and O, with a Ca/P ratio of 1.61 ± 0.08 and 1.60 ± 0.12 for crystalline and amorphous HA respectively. The TOF-SIMS showed that both the coated discs were composed of CaP, as indicated by ion signals from Ca and P species, whereas the titanium discs mainly displayed titanium and TiO species. Furthermore, Ti and TiO species were detected on the crystalline HA localized at specific spots on the surface, while minor scattered signals of Ti were observed for the amorphous HA. After C60 sputtering, the amorphous HA and titanium mainly showed Ti species, whereas the crystalline HA mainly showed TiO, indicating a thicker titanium oxide layer on the heat-treated sample. The XRD analysis showed only α-Ti for the control implant and amorphous HA, while the crystalline HA showed peaks at 2Θ = 31.8 and 32.9, indicating a crystalline phase of HA. The crystalline HA also showed peaks for titanium dioxide.

**Fig. 1:**
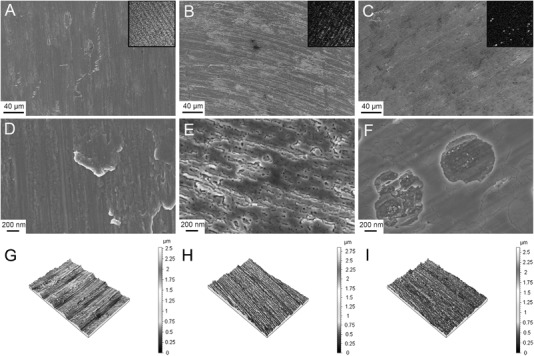
Surface characterization. The SEM micrographs show the machined titanium **a** and **d**, amorphous hydroxyapatite **b** and **e** and crystalline hydroxyapatite **c** and **f** surfaces at low and high magnification. The inserts in A–C show the TOF-SIMS signals for the corresponding surfaces. The 3D reconstructions of the interferometry analysis show the surface roughness of the machined titanium **g**, amorphous hydroxyapatite **h** and crystalline hydroxyapatite **i** surfaces

The quantitative topographical measurements showed similar values for the three different surfaces, with an *S*_a_ in the range of 0.23–0.27 µm, *S*_ci_ in the range of 1.50–1.61 and *S*_dr_ in the range of 2.86–4.27 % (Table 1). The three-dimensional reconstructions (Fig. 1) showed that all the different samples had a typical machined surface. This was further confirmed by the low-resolution SEM showing concentric machining patterns (Fig. 1). At high resolution, a clear difference was observed between the three surfaces, where the Ti showed a typical machined surface with ridges and smeared flakes. The amorphous HA showed a nano porous surface structure, while the crystalline HA appeared smoother with some cracks. Some coating had flaked off, revealing the Ti surface underneath.

**Table 1:** Topographical analysis of different roughness parameters (*S*_a_, *S*_ds_ and *S*_ci_) and the contact angle (*θ*) of the different surfaces

	Surface roughness			Contact angle
	*S*_a_ mean (SEM)	*S*_dr_ mean (SEM)	*S*_ci_ mean (SEM)	*θ* [°] (SEM)
Titanium	0.23 (0.009)	3.08 (0.18)	1.50 (0.021)	60.9 (0.8)
Amorphous HA	0.25 (0.008)	2.86 (0.18)	1.61 (0.038)	89.0 (4.9)
Crystalline HA	0.27 (0.002)	4.27 (0.24)	1.54 (0.012)	58.2 (2.0)

The data are presented as the mean (standard error of the mean, SEM)

The contact angle measurements revealed amorphous HA as the least hydrophilic surface of the three materials. Measured at 10 s after deposition of the droplet, the contact angles were titanium (60.9 ± 0.77), amorphous HA (89.1 ± 4.9) and crystalline HA (58.2 ± 2) (Table 1).

### Cytotoxicity

**Fig. 2:**
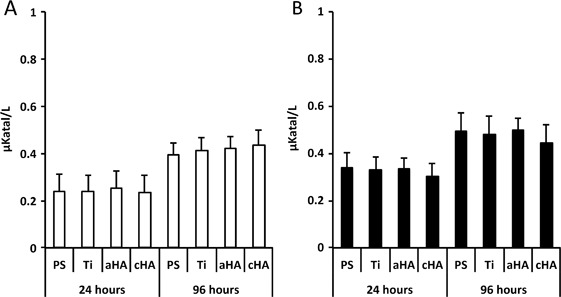
LD analysis. Release of lactate dehydrogenase (LD) from human monocytes at polystyrene (PS), titanium (Ti), amorphous HA (aHA) and crystalline HA (cHA) surfaces at 24 and 96 h. **a** Without the presence of lipopolysaccharide (LPS). **b** With the presence of 10 ng/ml of lipopolysaccharide (LPS). No statistically significant differences were found between the different surfaces, irrespective of LPS activation. The LD increase from 24 h to 96 h was statistically significant (*p* < 0.05) for all analyzed surfaces, both for non-LPS and for LPS-stimulated conditions. The data are presented as the mean + standard error of the mean

The lactate dehydrogenase (LD) analysis revealed significantly higher values at 96 h compared with 24 h, irrespective of implant type or LPS stimulation. Otherwise, there was no significant difference between the different implant types, either at 24 or at 96 h (Fig. 2).

### Cell counting

**Fig. 3:**
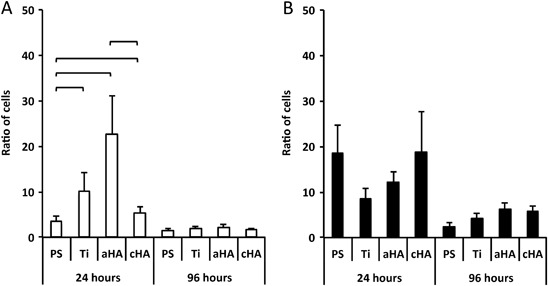
Ratio of implant-adherent cells in relation to cells in supernatant, at 24 and 96 h. **a** Without the presence of lipopolysaccharide (LPS). **b** With the presence of 10 ng/ml of lipopolysaccharide (LPS). Statistically significant differences (*p* < 0.05) between polystyrene (PS), titanium (Ti), amorphous HA (aHA) and crystalline HA (cHA) surfaces are indicated by bars. The data are presented as the mean + standard error of the mean

At 24 h, the total cell number ranged between 5 × 10^5^–6 × 10^5^ cells, without any differences between the surfaces and irrespective of LPS stimulation. However, in the non-LPS groups, the results revealed a significantly higher number of cells adhering to amorphous HA compared with PS. Moreover, the analysis revealed that amorphous HA had the lowest number of cells in the supernatant in comparison to other experimental groups. When the ratio of adherent cells to supernatant cells was determined, the highest ratio of adherent cells was demonstrated for amorphous HA in comparison to crystalline HA and PS (Fig. 3). After 96 h, all the experimental groups in the non-LPS condition revealed a reduction in the total cell number to a level range of 2.5 × 10^5^–3 × 10^5^ cells. On the other hand, the reduction in the total cell number was less pronounced in the LPS-stimulated cultures, where the total number of cells found in LPS cultures was about twice as high compared with non-LPS cultures, irrespective of the implant surface. At this time point (96 h), no major differences could be detected between the different surfaces, either for supernatant or for the implant-adherent cells.

### ELISA measurements

**Fig. 4:**
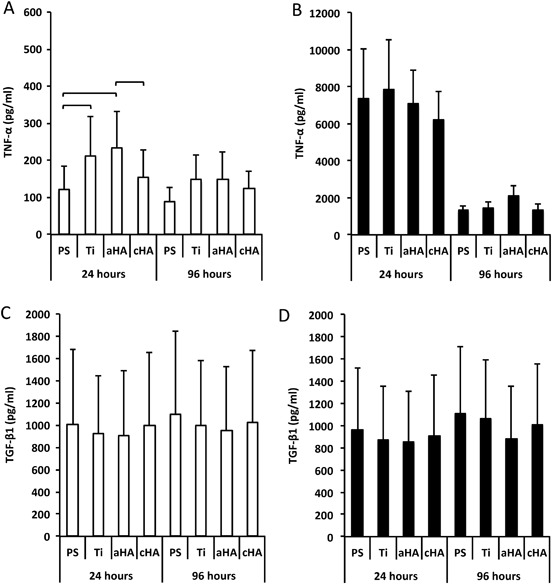
Enzyme-linked immunosorbent assay (ELISA). The analysis show the secretion of TNF-α **a** and **b** and TGF-β1 **c** and **d** from human monocytes cultured on polystyrene (PS), titanium (Ti), amorphous HA (aHA) and crystalline HA (cHA) at 24 and 96 h. The cells were cultured either without **a** and **c** or with **b** and **d** the presence of 10 ng/ml of lipopolysaccharide (LPS). Statistically significant differences (*p* < 0.05) between the different surfaces are indicated by bars. The data are presented as the mean + standard error of the mean

With respect to TNF-α secretion in the non-LPS condition, both the amorphous HA and Ti showed significantly higher levels compared with the control PS surface at 24 h (Fig. 4a). Furthermore, the amorphous HA demonstrated a significantly higher TNF-α secretion when compared with crystalline HA. With LPS stimulation, approximately 30 times higher secretion was found for all groups compared with non-LPS, with no significant differences detected between the different surfaces (Fig. 4b). At 96 h in the non-LPS groups, despite the slight general reduction in the TNF-α concentration, no major differences could be found between the different surfaces (Fig. 4a). A significant reduction was found for all surfaces with LPS stimulation after 96 h of culture, although it was still higher compared with the levels found in the non-LPS cultures (Fig. 4b).

Regarding TGF-β1, the secretion of this growth factor was demonstrated with a mean range of 900–1000 pg/ml for all groups, irrespective of time point or LPS stimulation (Figs. 4c and d). No significant differences could be found between the surfaces at any of the evaluated time points, either with or without LPS stimulation (Figs. 4c and d).

With the present assay, no measurable BMP-2 secretion was detected for any of the analyzed surfaces and at any of the analyzed time points (detection limit 62.5 pg/ml).

## Discussion

There is general consensus that HA promotes osseointegration and this is supported by numerous clinical and preclinical reports [24–27]. During osseointegration, the early inflammatory process is tightly linked to bone regeneration and remodeling at the bone-implant interface [28]. Since monocytes/macrophages are among the first cells to be found at the interface, a question has been raised about whether these cells convey osteogenic signals when encountering surfaces with a bone-promoting effect. To investigate this hypothesis, we analyzed the secretion of the pro-osteogenic factors, BMP-2 and TGF-β1, from monocytes cultured on titanium and titanium with amorphous or crystalline thin HA coatings.

In the present study, no BMP-2 secretion by primary human monocytes/macrophages was found at any of the investigated surfaces. This finding is in partial agreement with another report, using murine J774A.1 macrophages cultured on plasma-sprayed HA [29]. In the latter study, although BMP-2 was detected at the RNA level in the cell line, BMP-2 secretion was not detected at the protein level. Furthermore, it has also been revealed that, when primary human monocytes are triggered toward the regenerative pathway, i.e., alternative macrophage (M2) activation with IL-4, the M2 macrophages did not secrete BMP-2, either on PS or on machined or oxidized titanium surfaces [22]. Contrary to these studies, murine J774A.1 macrophages both expressed and secreted BMP-2, with higher BMP-2 secretion detected at HA and PS surfaces compared with titanium [30]. The contradictions between these findings may reflect the different types of macrophage used, whether they were primary cells or cell lines. This assumption is supported by findings from two separate studies investigating BMP-2 gene expression in different cell lines and/or primary cells [21, 31]. In the first study, although the BMP-2 gene expression of different macrophage cell lines was confirmed, the gene expression of BMP-2 was not determined in primary human monocytes [31]. In the second study, the gene expression of BMP-2 was detected in three different macrophage cell lines: human THP-1 and mouse RAW264.1 and J774A.1 cell lines, whereas primary human monocytes were not investigated [21]. Although it has been confirmed that macrophage cell lines express and possibly secrete BMP-2, there is still no clear evidence that primary human monocytes convey osteogenic potential via the secretion of BMP-2.

In the present study, it was shown that the pro-osteogenic factor, TGF-β1, but not BMP-2, was secreted by primary human monocytes/macrophages on all evaluated surfaces and irrespective of LPS. This finding is in agreement with observations showing no major differences in TGF-β secretion from a J774A.1 macrophage cell line cultured on plasma-sprayed HA and titanium surfaces [29]. In line with these observations, the gene expression of TGF-β was not affected by different surface topography when a J774A.1 macrophage cell line was cultured on polished or grit-blasted titanium surfaces [30]. Moreover, in an in vivo subcutaneous mice model, although the gene expression of TGF-β was significantly higher at titanium surfaces with different groove patterns compared with smooth ones, the analysis of TGF-β secretion by ELISA revealed no differences between the different groove patterns and the smooth control [32]. Taken together, these findings indicate that monocytes/macrophages are capable of conveying pro-osteogenic signals via the secretion of TGF-β and that TGF-β secretion does not appear to be affected by the surface properties of the implants, or by the presence of strong pro-inflammatory stimuli.

One of the main findings in this study was that, in the absence of strong inflammatory stimuli (LPS), the secretion of TNF-α was modulated by the different properties of the HA coating. High TNF-α secretion was found in response to titanium and amorphous HA but not the crystalline HA. The finding of a transient increase in TNF-α gene expression and secretion in response to machined titanium surfaces has been reported in several in vitro and in vivo studies [29, 33–35]. On the other hand, less information is available on TNF-α secretion in response to HA surfaces, especially in relation to differences in crystallinity and phase composition. Titanium and titanium alloy discs with a 20–100 nm crystalline CaP layer significantly reduced the gene expression of TNF-α and showed a lower trend towards TNF-α secretion in a RAW 264.7 macrophage cell line compared with discs without the crystalline CaP [33]. These findings suggest that the bone-enhancing effect of crystalline HA [8, 24] and CaP-based coatings [5] may be partly mediated via the reduction of pro-inflammatory TNF-α. Hitherto, the role of TNF-α secretion in bone regeneration and osseointegration remains controversial. An in vitro study revealed that classically activated monocyte CM, containing a high level of TNF-α, enhanced osteogenic gene expression in human MSCs [22]. Further, a positive correlation was found between the expression of TNF-α and bone-formation genes (ALP and OC) during defect healing augmented with a CaP-based bone substitute [36]. However, the downregulation of TNF-α expression in the implant-adherent cells is associated with enhanced osteogenic gene expression and the promotion of bone formation and implant stability at the interface between modified titanium implants and bone [37]. For this reason, the possibility cannot be excluded that a reduction in TNF-α in cells adhering to implant surfaces provides a boosting mechanism to enhance the osseointegration process. The reduction in TNF-α, together with the availability of a pro-osteogenic signal, such as TGF-β1, at the crystalline HA, may, in part, explain the enhanced osseointegration at the crystalline HA-coated implants [8, 24].

The present findings are in partial agreement with previous results in a rat soft-tissue model, using identical amorphous and crystalline HA-coated titanium implants [15]. In the latter study, a lower inflammatory response was demonstrated for both types of HA surface and a smaller number of cells adhered to amorphous HA compared with titanium, as determined by the amount of DNA in the implant-adherent cells [15]. Furthermore, relatively lower cytotoxicity, as measured by LD analysis, and lower MCP-1 secretion were detected in response to amorphous HA and, to a lesser extent, at the crystalline HA, when both were compared with titanium [15]. The reason for the discrepancy between the present in vitro findings and the soft-tissue findings cannot currently be determined. Further, it remains to be established whether the inflammatory response in bone differs between the amorphous and crystalline HA coatings.

In order to explore plausible reasons for the lower TNF-α secretion at the crystalline HA, a thorough surface characterization was performed with the emphasis on surface parameters that may affect the adhesion and/or spread of the monocytes. In the present study, the ratio of implant-adherent monocytes in relation to supernatant ones was higher at the amorphous HA and titanium surfaces, when compared with PS. Moreover, the crystalline HA revealed a lower ratio compared with amorphous HA. The material characterization demonstrated a relatively higher hydrophobicity for the amorphous HA compared with the other surfaces. The reports of cell adhesion to surfaces with different wettability suggest better adhesion to hydrophilic surfaces [38, 39]. In contrast, hydrophobic surfaces have been reported to favor the adhesion of monocytes [40, 41]. Since the adhesion of the cells is mediated via the adsorbed protein layer, the amorphous HA may have influenced a higher degree of protein adsorption than the other surfaces. For instance, it has been shown that albumin preferentially adsorbs to amorphous HA, with an adsorption rate correlating inversely to the HA crystallinity [42, 43]. One reason for an increase in hydrophobicity could be related to surface contamination. Surface contamination has been shown to be an important factor for wettability, where higher surface contamination leads to lower wettability [44]. However, the combination of XPS and TOF-SIMS showed similar surface contaminants on the current discs.

The nanotopography of the surface is another parameter influencing wettability [45]. In the present study, whereas all surfaces had similar micro-scale roughness and were regarded as smooth (*S*_a_ around 0.2–0.3 µm), they differed at the nano-scale level. The amorphous HA, which induced the highest adhesion of monocytes and an increase in TNF-α secretion, also revealed a nano-topography, in the form of porous structures. This finding implies an association between nano-topography and increased pro-inflammatory activity. In contrast, a recent in vivo study has shown that titanium implants with controlled nano-topography (80 nm hemispherical protrusions) resulted in the lower recruitment of macrophages and lower TNF-α expression in the implant-adherent cells compared with implants without nano-topography [46]. One plausible explanation is that the effect of nano-topography on macrophages is dependent on the shape and size of the nano-features. This assumption is supported by an in vitro study showing that a murine-macrophage (RAW264.7) increased the expression of TNF-α in response to surfaces with a groove-shaped nano-topography [32]. Another plausible explanation is that the macrophage response is also dependent on other surface characteristics, including surface chemistry, regardless of whether it is HA, as in the present study, or titanium, as in the previous in vivo study [46]. The influence of chemistry in relation to nano-topography has been previously addressed in an in vivo study, where nano-topography did not modify the in vivo bone response to the same extent as the chemistry did [47]. This implies that the early inflammatory response is likely to be influenced by the combination of different surface properties, which highlights the importance of thorough surface characterization.

## Conclusions

The present findings show that differences in the phase composition, nano-scale topography and/or wettability of the HA thin coating are associated with different early monocyte responses. It is concluded that, under in vitro conditions and in the absence of strong pro-inflammatory stimuli, crystalline hydroxyapatite elicits a lower inflammatory cell response compared with amorphous hydroxyapatite, while tentatively not affecting the osteogenic capacity of human monocytes.
